# Human biting rhythm of *Anopheles gambiae* Giles, 1902 (Diptera: Culicidae) and sleeping behaviour of pregnant women in a lagoon area in Southern Benin

**DOI:** 10.1186/s13104-021-05615-7

**Published:** 2021-05-22

**Authors:** Armel Djènontin, Aziz Bouraima, Christophe Soares, Seun Egbinola, Gilles Cottrell

**Affiliations:** 1 Centre de Recherche Entomologique de Cotonou (CREC), 06 BP 2604 Cotonou, Bénin; 2 Centre de Recherche pour la lutte contre les Maladies Infectieuses Tropicales (CReMIT), Université d’Abomey-Calavi (UAC), BP 526 Cotonou, Bénin; 3 Heartland Alliance, Lagos, Nigeria; 4 UMR216-MERIT, Institut de Recherche pour le Développement, 75006 Paris, France

**Keywords:** *Anopheles coluzzi*, Biting rhythm, Malaria, Prevention, Pregnant women

## Abstract

**Objective:**

In the framework of EVALMOUS study aiming to assess the use and effectiveness of mosquito nets by pregnant women and other members of their household in a lagoon area in southern Benin, the behaviour of pregnant women relative to the time they go to bed using the net were recorded. Malaria vectors biting rhythm, *Plasmodium falciparum* infection and insecticide resistance genes in malaria vectors were also determined.

**Results:**

Overall, 3848 females of *Anopheles gambiae s. l* were collected and 280 pregnant women responded to the survey. Almost all *Anopheles gambiae s. l.* tested were *Anopheles coluzzi* Coetzee and Wilkerson 2013 (Diptera: Culicidae). The CSP index in malaria vector was 1.85% and the allelic frequency of *kdr* gene was 74.4%. Around 90% of bites and *Plasmodium falciparum* Welch, 1897 (Haemosporida: Plasmodiidae) transmission occurred between 10 p.m. and 6 a.m., which coincides with the period when more than 80% of pregnant women were under bednet. Despite a slight early evening and early morning biting activity of malaria vectors in the study area, the good use of nets might remain a useful protection tool against mosquito biting and malaria transmission.

**Supplementary Information:**

The online version contains supplementary material available at 10.1186/s13104-021-05615-7.

## Introduction

Since 2000, a significant expansion of financial support and scale‐up of malaria control tools were observed [1]. Progress in terms of reduction in malaria cases and deaths followed this scale‐up of malaria control tools. Despite this, malaria remains a major public health concern in Africa, with an estimated 213 million cases and 3,80,000 deaths in 2018 [2], probably due to resistance in parasite and vectors and changes in vectors behaviour. In Benin, malaria cases increased from 10,44,235 and 1869 deaths to 17,68,450 malaria cases and 2138 deaths between 2014 and 2018.

In many malaria endemic areas including Benin, Long Lasting Insecticidal Treated Nets (LLITNs) and Indoor Residual Spraying (IRS) represent the main interventions for malaria vector control [2] as recommended by World Health Organisation (WHO). These indoor insecticide‐based methods and LLITNs depend on various factors among which vectors behaviour (such as human biting rythm and endo/exophagy), human behaviour (e.g. sleeping behaviour) and insecticide resistance mechanisms in vectors. Insecticide resistance is well documented in malaria vectors in malaria endemic countries [3, 4]. Furthermore, numerous studies across endemic countries reported behavioural changes of malaria vectors [5–7]. Early morning biting activity of *Anopheles (Cellia) funestus* Giles, 1900 (Diptera: Culicidae) following the universal coverage of LLITNs was reported in Southern Benin and authors stated that these findings might have direct consequences for malaria control in Africa [5]. Indeed, this vector could bite and transmit the parasite at times when people are not under LLITNs. EVALMOUS study assessed the use and the effectiveness of mosquito nets by pregnant women and other members of their household in preventing malaria [8]. This study was carried out in Sô-Ava district, a lagoon area with high risk for malaria vectors proliferation [9]. Entomological surveys and questionnaire were carried out to assess malaria vectors human biting activity and the sleeping behaviour of pregnant women.

## Main text

### Methods

#### Study area and type of study

EVALMOUS study was carried out in 15 villages of Sô‐Ava district (6°28′ 00″ N 2° 25′ 00″ E) located in Nokoue Lake in Southern Benin. Sô‐Ava is one of the eight districts of Atlantic department with about 1,00,000 inhabitants and a surface area of 218 km^2^. The present study is an observational cross-sectional study nested in EVALMOUS study. Inclusion and exclusion criteria of EVALMOUS study are described in Hounkonnou et al. 2018 [8]. Any household in the EVALMOUS study that accepted overnight mosquito collection was eligible (no household refused).

#### Mosquito collection

Mosquito collection was carried out in 15 villages selected for EVALMOUS study, using human landing catches method (HLCs) as described by Djènontin et al., 2010 [10]. In each village, mosquito collection was carried out both indoors and outdoors of 3 human cases for 1 night (90 collection sites in all) during the rainy season between July and August 2016. Mosquitos were collected from 7 p.m. to 9 a.m. Collector teams were rotated among the collection points to minimize sampling bias.

#### Laboratory analysis

Mosquitoes were identified to species level using morphological criteria according to the identification keys [11, 12]. All mosquitoes identified as *Anopheles gambiae s. l.* were stored in individual tubes with silica gel and preserved at − 20 °C in the laboratory.

Heads and thoraxes of anopheline females were tested by enzyme‐linked immunosorbent assays (ELISA) for detection of *Plasmodium falciparum* circumsporozoite protein (CSP), as previously described [13].

Genomic DNA was extracted from the abdomen of all mosquito using the extraction buffer Livak [14]. The species was then identified by diagnostic Polymerase Chain Reaction (PCR) using the Scott protocol [15] and molecular characterization according to Santolamazza protocol [16, 17]. The knockdown resistance mutation (*kdr*‐west, L1014F) was detected by Taqman allelic discrimination assays as described by Bass et al.[18].

#### Pregnant women sleeping behaviour and bed net use

Data regarding the time at which each pregnant woman entered and exited their house, the time each bed net user entered and exited their sleeping space the night preceding the survey were collected by questionnaire.

#### Data analysis

Every hour from 7 p.m. to 9 a.m, the proportion of malaria vectors collected indoors was determined, as well as the proportion of pregnant women under bed nets at each hour from 7 p.m. to 9 a.m the night preceding the survey.

The mosquito collection period was divided into 3 different timeframes (7 p.m.–10 p.m.; 10 p.m.–6 a.m.; 6 a.m.–9 a.m.). The proportion of mosquito collected at each timeframe (aggressiveness) was calculated, as well as the 95% confidence interval.

CSP index in malaria vectors *Anopheles gambiae s.l.* in each village was calculated as the number of positive vector divided by the total tested. Endophagy was calculated as the number of vectors collected indoors divided by the total collected. The 95% confidence interval of the CSP index and endophagy were also estimated. Allelic frequency of *kdr* gene mutation in *Anopheles gambiae s.l.* at each period was calculated. Proportions were compared using Chi-square tests.

### Results

#### Human biting rhythm and pregnant women sleeping behaviour

**Fig. 1:**
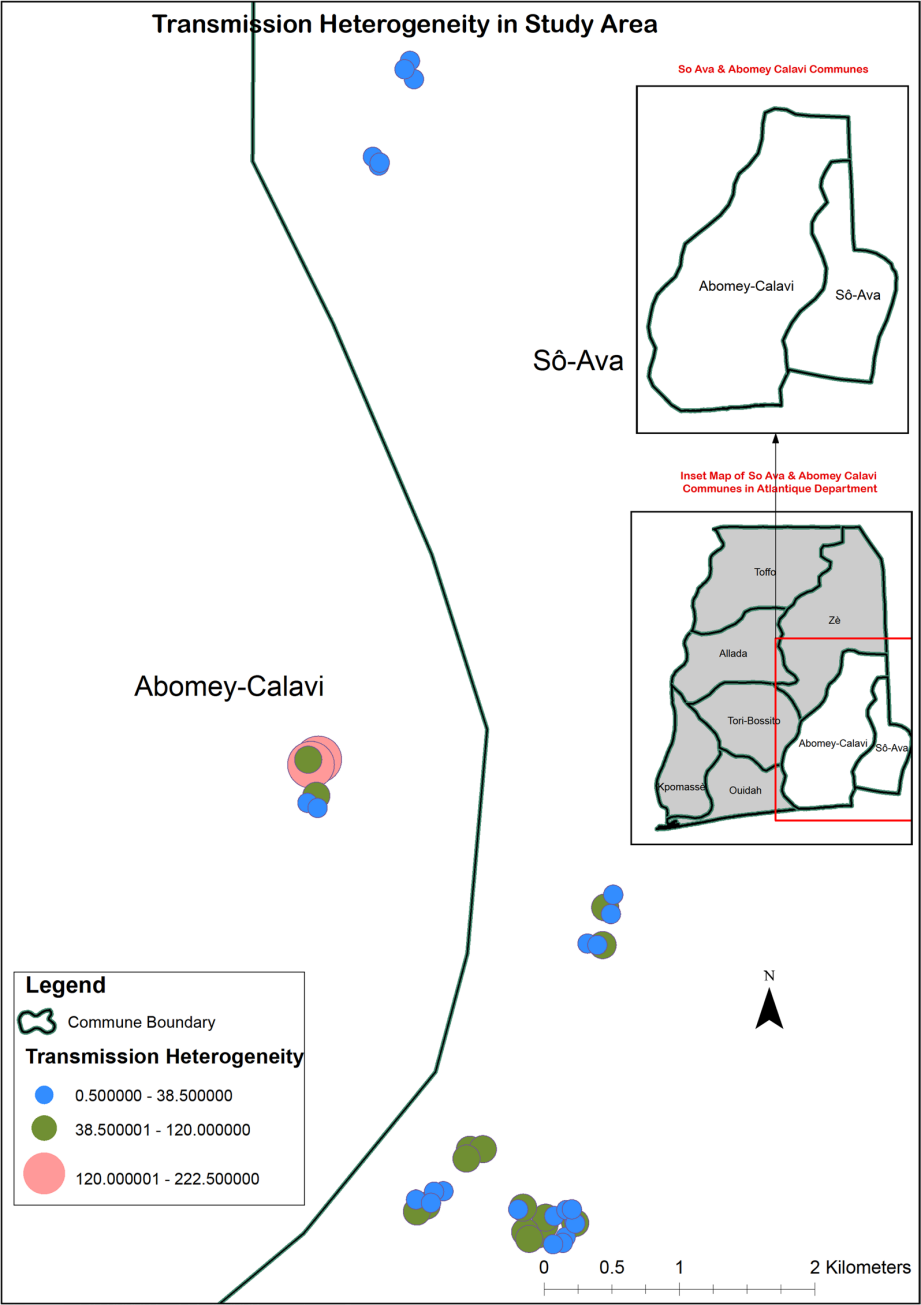
Spatial heterogeneity of malaria vectors abundance in the study area

Overall, 10,285 mosquitoes were caught of which 334 were *Anopheles (Cellia) pharoensis* Theobald, 1901 (Diptera: Culicidae) and 3848 were *Anopheles gambiae s. l.* The distribution by village of *Anopheles gambiae s. l.* is shown the Additional file 1: Table S1. A high spatial heterogeneity of abundance malaria vectors was observed (Fig. 1). The number of females of *Anopheles gambiae s.l.* collected ranged from 14 (corresponding to 2.33 bites per person per night in Awomey-Gbèkpa village) to 1114 (corresponding to 185.66 bites per person per night in Todo village). Respectively 1.3%, 90.7% and 8.0% of mosquito’s aggressiveness occurred during the timeframes 7 p.m–10 p.m.; 10 p.m–6 a.m. and 6 a.m–9 a.m. (Additional file 2: Table S2).

**Fig. 2:**
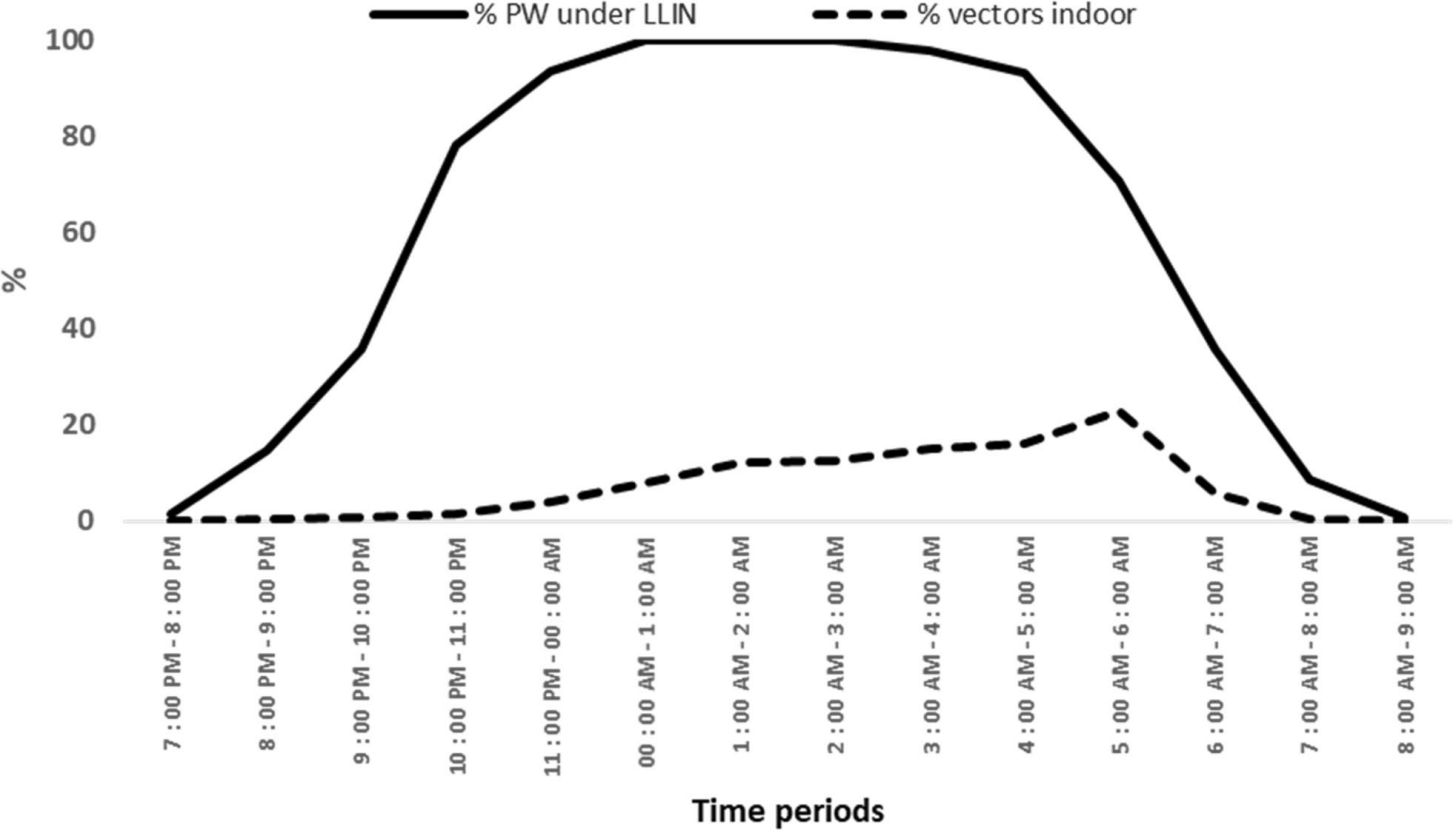
Proportion of pregnant women (PW) under bed nets and proportion of vectors collected indoor per period

A total of 280 pregnant women responded to the survey. The proportion of pregnant women under bed net from early evening to early morning ranged from around 1% (8 a.m–9 a.m.) to 100% (12 a.m–3 a.m.) (Fig. 2). From 10 p.m. to 6 a.m., where around 90% of aggressiveness and transmission occurred in the study area during the survey, 85.5% of pregnant women were under bed net (Fig. 2).

#### Malaria vectors species and *Plasmodium falciparum* infection

From the 3848 females of *Anopheles gambiae s. l.* collected, 1383 were randomly selected and successfully identified at species level using PCR. Almost all *Anopheles gambiae s. l.* tested were *Anopheles coluzzi* except 2 which were *Anopheles melas* (Theobald 1903) (Diptera: Culicidae).

The overall CSP index was 1.85 ± 0.01% (Table 1). CSP antigen index showed a spatial heterogeneity ranging from 0% (in Awomey-Gbèkpa, Awomey-Gblon and Nonhouéto villages) to 5.77 ± 0.88% in Hounhouè village. Respectively 1.41%, 94.37% and 4.23% of *Plasmodium falciparum* transmission occurred during the periods 7 p.m–10 p.m.; 10 p.m–6 a.m. and 6 a.m–9 a.m. (Table 1).

**Table 1:** CSP antigen index in malaria vectors and *Plasmodium falciparum* transmission both indoor and outdoor per period with 95% CI

	N vector tested	N vectors CSP^+^	CSP^+^ rate (%)	95%CI	(%) Transmission
7 p.m–10 pm					
Indoor	34	1	2.94	0.97	1.41
Outdoor	17	0	0.00	0.00	0.00
Total	51	1	1.96	0.53	1.41
10 pm–6am					
Indoor	1998	39	1.95	0.01	54.93
Outdoor	1492	28	1.88	0.02	39.44
Total	3490	67	1.92	0.01	94.37
6 am–9 am					
Indoor	139	1	0.72	0.12	1.41
Outdoor	168	2	1.19	0.13	2.82
Total	307	3	0.98	0.06	4.23
Total					
Indoor	2171	41	1.89	0.01	57.75
Outdoor	1677	30	1.79	0.02	42.25
Total	3848	71	1.85	0.01	100

The endophagy, was estimated on the total sample at 56.4 ± 0.01%. Endophagy was significantly higher (df = 1, p = 0.005) at the early evening (7 p.m–10 p.m. 66.7 ± 12.9%) compared to the early morning (6 a.m–9 a.m., 45.2 ± 5.6%), (Additional file 2: Table S2).

#### Allelic frequency of *kdr* gene in *Anopheles gambiae s.l.*

The allelic frequency of *kdr* gene in *Anopheles gambiae s. l.* collected in the villages was 74.4%. No significant difference (df = 1, p = 0.186) was observed between the allelic frequency of this gene indoors (75.4%) and outdoors (73.3%) on the total sample (Additional file 3: Table S3). However, the allelic frequency of *kdr* gene in *Anopheles gambiae s. l.* biting human indoors early morning (6 a.m–9 a.m.), 82.0 [77.5–86.5] % seems higher than that of *Anopheles gambiae s. l.* biting human outdoors during the same period (72.8 [68.0–77.5] %), but difference was not significant (df = 1, p = 0.816).

#### Discussion

The present study reported precious data, seen that such data on aggressiveness rhythm and human behaviour collected simultaneously in the same setting are rare. Data shows that despite a slight early evening and early morning biting activity of malaria vectors, more than 90% of malaria vectors bites and *Plasmodium falciparum* transmission occurred between 10 p.m. and 6 a.m., a period at which over 80% of pregnant women were under bed net in the study area. The main malaria vector in the study area was *Anopheles coluzzi* with a CSP index and an allelic frequency of *kdr* gene equal respectively to 1.9% and 74.4%.

The early evening and early morning biting activity reported in this study are consistent with recent findings on *Anopheles funestus* in Southern Benin [5]. Despite this unusual malaria vectors biting activity in the study area, bed net remains a useful protection tool against mosquito biting since more than 90% of bites and *Plasmodium falciparum* transmission occurred between 10 p.m. and 6 a.m., period during which most pregnant women were in bed under bed net. Analysing data collected in 2011 on behavioral interactions between *Anopheles funestus* and humans in two villages of southern Benin, Moiroux et al. showed that the true average personal protection of LLITNs remained very high (> 80%), despite the change in this vector biting peak [19]. The critical challenge for malaria control in the study area seems to remain the good use, bio efficacy and the physical integrity of LLITNs. Indeed, as reported by Hounkonnou et al., around 90% of pregnant women in the study area used bed net, but less than 10% of bed net used by pregnant women attained WHO bio-efficacy criteria [8]. It is also important to note that in the present study, aggressiveness and transmission had a very high spatial heterogeneity ranging from 2.33 bites (0 infected bite) per person per night in Awomey-Gbèkpa village to 185.66 bites (4 infected bites) per person per night in Todo village. These findings highlight the existence of local foci of transmission in the study area where the aggressiveness and transmission remain high even in the timeframe 6 a.m.–9 a.m. when majority of pregnant women were not under LLITNs. In Todo village, aggressiveness and transmission were respectively 14.85 bites per person and 0.32 infected bites per person during this timeframe).

The study area is a natural lake influenced by the Atlantic Ocean and previous studies carried out 2 decades ago in neighbouring lagoon areas reported that *Anopheles melas* represents 80% of *Anopheles gambiae s.l.* [20, 21]. The low *Anopheles melas* proportion reported in the present study was recently reported in the same district [9], and could be due to its urbanized nature which has probably led to the proliferation of breeding sites of *Anopheles coluzzii* at the detriment of *Anopheles melas* [9, 20]. Since *Anopheles melas* is known to have a sporozoitic index significantly lower than that of *Anopheles coluzzii* [20–22], the relative proliferation of breeding sites of *Anopheles coluzzii* is a potential health concern considering malaria transmission in this area. However, subsequent increase in malaria transmission in this area have been masked by the large scale implementation of vectors control tools (e.g. Long Lasting Insecticidal Nets) [9]. A recent study carried out in this study area showed a general high possession of bed nets by the pregnant women with more than 8 out of 10 women using it [8].

The allelic frequency of *kdr* gene in *Anopheles coluzzii* reported in the present study is consistent with that reported recently in neighbouring villages [9] and was lower than 100% observed in Cotonou, a neighbouring district. As fishing is the most important economic activity in the study area, there is a lower use of insecticides linked to agriculture. The insecticide resistance selection in this area could be due to the domestic use of insecticide for vectors control [9]. In addition, an increase in the activity of vegetable farming in neighbouring districts [23] has led to the use of insecticides in an improper manner to control vegetable pests and pesticide residues from these areas can also contribute to insecticide resistance selection in the study area [9].

### Conclusion

The present study revealed a non-negligible early evening and early morning biting activity of malaria vectors in the study area. However, the major part of transmission occurs at a period when more than 80% of pregnant women were on bed under bed net. Findings of this study indicate that in the study area, there is no argument indicating that the good use of LLITNs do not provide to pregnant protection against mosquito biting and malaria transmission. Then the use of LLITNs with good physical integrity and bio-efficacy have to be promoted among pregnant women despite changes in vectors aggressiveness rhythm.

## Limitations

This study design is an observational cross-sectional and mosquito collection was carried out in 3 different points only for one night due to logistic reasons. This could be considered as a limitation, and a longitudinal design may have brought a higher level of evidence. However, the data collected is precious because such data; aggressiveness rhythm and human behaviour collected simultaneously in the same setting, are rare. Essentially, it is of crucial importance to monitor these indicators at a high scale and with a higher frequency.

## Supplementary Information

**Additional file 1:** **Table S1.** Number of malaria vectors caught and CSP antigen index per village with 95% CI.

**Additional file 2:** **Table S2.** Endophagy according to the periods with 95% CI.

**Additional file 3:** **Table S3.** Resistance gene *kdr*_w_ in malaria vectors according to the periods.

## Data Availability

The datasets during and/or analysed during the current study are available from the corresponding author on reasonable request.

## Abbreviations

CSP: Circumsporozoite protein
ELISA: Enzyme‐linked immunosorbent assays
HLCs: Human landing catches
IRS: Indoor residual spraying
*Kdr*: Knockdown resistance
LLITNs: Long lasting insecticidal treated nets
PCR: Polymerase chain reaction
*s. l.*: Sensu lato
WHO: World Health Organisation
