# Supplementary material for: Human biting rhythm of Anopheles gambiae Giles, 1902 (Diptera: Culicidae) and sleeping behaviour of pregnant women in a lagoon area in Southern Benin
Source: BMC Res Notes. 2021 May 22;14:200. doi: 10.1186/s13104-021-05615-7 (PMC8141146; doi:10.1186/s13104-021-05615-7)
Supplement: Supplementary file 1 — Additional file 1: Table S1. Number of malaria vectors caught and CSP antigen index per village with 95% CI. [file 13104_2021_5615_MOESM1_ESM.docx]

**Table S1: Number of malaria vectors caught and CSP antigen index per village with 95% CI**
