# Supplementary material for: Human biting rhythm of Anopheles gambiae Giles, 1902 (Diptera: Culicidae) and sleeping behaviour of pregnant women in a lagoon area in Southern Benin
Source: BMC Res Notes. 2021 May 22;14:200. doi: 10.1186/s13104-021-05615-7 (PMC8141146; doi:10.1186/s13104-021-05615-7)
Supplement: Supplementary file 3 — Additional file 3: Table S3. Resistance gene kdrw in malaria vectors according to the periods. [file 13104_2021_5615_MOESM3_ESM.docx]

**Table S3: Resistance gene *kdr*_w_ in malaria vectors according to the periods**
